# Wireless Body Area Network (WBAN)-Based Telemedicine for Emergency Care

**DOI:** 10.3390/s20072153

**Published:** 2020-04-10

**Authors:** Latha R, Vetrivelan P

**Affiliations:** School of Electronics Engineering, Vellore Institute of Technology, Chennai 600127, India; latha.r2015@vit.ac.in

**Keywords:** wireless body area network, prior probability, evidence, immediate consultation, posterior probability

## Abstract

This paper is a collection of telemedicine techniques used by wireless body area networks (WBANs) for emergency conditions. Furthermore, Bayes’ theorem is proposed for predicting emergency conditions. With prior knowledge, the posterior probability can be found along with the observed evidence. The probability of sending emergency messages can be determined using Bayes’ theorem with the likelihood evidence. It can be viewed as medical decision-making, since diagnosis conditions such as emergency monitoring, delay-sensitive monitoring, and general monitoring are analyzed with its network characteristics, including data rate, cost, packet loss rate, latency, and jitter. This paper explains the network model with 16 variables, with one describing immediate consultation, as well as another three describing emergency monitoring, delay-sensitive monitoring, and general monitoring. The remaining 12 variables are observations related to latency, cost, packet loss rate, data rate, and jitter.

## 1. Introduction

With similarities to first come first serve (FCFS), traditional transmission in healthcare has local data processing units which prioritize, according to their time slots, the treatment of patients with emergency conditions. The prioritization illustrates the importance of a particular node from energy dissipation and the amount of time taken after transmission [1]. An algorithm is designed based on fitness priority chosen, which clearly signifies the fitness which is higher. The higher fitness is chosen to have a lower waiting time for data packets. The scheduling packet is modeled according to the priority, and many multi-level schedulers are used for transmission [2]. Wireless body area networks (WBANs) help in monitoring health activities regardless of patient location and only teleconsultation is needed, thus reducing the visiting hours of doctors. WBANs monitor humans’ e-health as a key element [3], but WBAN has many challenges with respect to data, network, interoperability, security, and scalability [4]. Wearable sensors help in collecting information about the physiological parameters of the patient, which are to the doctor at the medical center to analyze the information [5]. The WBAN must transfer patient information securely, quickly, and efficiently, without any change in data, and the energy consumption must also be low [6]. The IEEE 802.15.6 WBAN standard has measurements on channel modes, while it also includes the mobility of nodes and effects caused by human interaction [6]. Medical WBAN ensures the early detection of diseases, thus resulting in the prevention of illness and a decrease in healthcare cost [6,7]. WBAN is capable of providing ubiquitous healthcare services and maintains these records in servers [7]. Because of WBAN, there are many inventions which are easily usable technologies for monitoring the health of patients [8]. This pervasive application of WBAN is the most easily available technology for patients [9], since the WBAN nodes help in collecting and storing the patient’s data, offering healthcare services [10]. WBAN gives the real-time status of the patient, which provides the best monitoring without affecting daily activities [11], and it also sends health-related information to the doctor [12]. The IEEE 802.15.6 standard is suitable mainly for WBAN requirements since WBAN is widely used in telemedicine, as well as in remote monitoring of patients [13]. Through WBAN applications, the status of the patient, events of the patient, and their health are noted down [14]. This technology can revolutionize patient health monitoring in the future, allowing easy access to the patient’s condition from any place.

Physiological data are collected through WBAN nodes from the patient and transmitted to the sink [15]; thus, WBAN helps in the continuous monitoring of the patient and updates the status of the patient to a remote doctor [16]. Wearable equipment on the market now allows for providing users improved healthcare service [17]. In addition, WBAN provides the miniaturization of sensor nodes, which helps in the constant monitoring of remote patients [18]. The placement of relay nodes and the quality of service (QoS) requirement of medical signals result in a reliable and connected WBAN [19,20]. WBAN in healthcare must be secured through a robust authentication scheme [21], and these sensor readings may be inaccurate due to some faults, resulting in anomalous detection schemes [22]. WBAN network topology and cross-layer optimization were discussed in References [23,24], which optimized cost effectiveness through a multi-objective cost function. In Reference [25], a novel convergecast strategy, especially for WBAN, was designed using a delay-tolerant network and wireless sensor network in the OMNeT++ framework. In Reference [26], an authentication scheme for a medical body area network was designed, and its reliability was demonstrated. In Reference [27], authentication schemes for BAN were designed. In References [28,29], wearables that non-invasively detect drugs were surveyed with technologies for precision health and monitoring. In Reference [30], a telemedicine application using IEEE 802.15.6 MAC and IEEE 802.15.4 PHY was demonstrated, and, in Reference [31], the WBAN node energy was restrained.

A Bayesian network is a directed acyclic graph (DAG) with a set of random variables called nodes, and these nodes have direct links to the conditional probability table (CPT). Bayes networks are known to be graphical models which are probabilistic and based on expert knowledge derived from datasets. They are suitable for decision-making concerning uncertainty, and these networks are also called decision networks, causal networks, or belief networks, which depend on multiple events. Here, the real-time relationships are always probabilistic, and these networks represent probabilistic relations with multiple events. The node is represented as a hypothesis having at least two values, which are possible probabilities. The arrows give the conditional independencies of relationships between the state of node and its probability distribution and the other node to which it is connected. Decision theory is the combination of utility and probability theory, in which the nodes exhibit causal relationships and conditional independence between nodes. Bayes nets are directed graphical models representing hierarchical Bayesian models, and these networks find the probability of an uncertain cause with the help of observed evidence. As these Bayesian networks give a causal probabilistic relationship, the probability of the evidence can be found easily. The link between nodes gives the probabilistic relationship between them. Only loops can be formed, whereas cycles are not permitted in the graph. These networks mix probability with the values of measurement, and they also help in computing the given evidence. Bayes nets are also called belief nets or decision nets, and they help in optimizing decisions, thereby allowing probabilities to be updated. By using Bayes’ rule, there is no need for exact probabilities, but causal conditional probabilities are estimated easily and are applied in diagnosis, prediction, modeling, monitoring, and alerting.

## 2. Related Work

Agrahari analyzed a dynamic Bayesian network dealing with uncertainty and stochastic behavior, which helps in treating patients through telemedicine [31]. In telemedicine, the data are provided by the patient through sensors, and the diagnosis is confirmed or denied by the physician. The smart agent helps in predicting and correcting diagnosis. Smart agents improve the physician’s diagnosis. Bellot dealt with a Bayesian network, based on multi-agent systems which provide diagnosis decisions for a telemedicine framework [32]. This model was based on analyzing the probabilities of physiological indices, which included both a Bayesian and a multi-agent system. The framework for remote diagnosis was designed with the Bayesian inference for prediction and, therefore, the network model was estimated, tested, and verified through Bayesian statistics [33]. Here, the causal paths, uncertainty information, and prior knowledge were evaluated for hypotheses, and those approaches related to constraint-based and score-based methods were found for independent and dependent relationships in the graph [34]. Hill climbing, genetic algorithms, and tabu search are some of the heuristic search techniques used for DAGs. A Bayesian network was trained with Eigen genes, and the arc link conditional dependencies [35] were evaluated accurately for conditional probability density functions. These Bayesian networks are known for their predictions, as well as their interpretability [36], and they are helpful in making decisions, with cause-and-effect relationships, for statistical analysis. The connections in the medical data are interpreted, representing causality and uncertainty, and these Bayesian medical network models are quantified through mutually exclusive states. Their dependencies are noted in a CPT [37]. Adding evidence changes the probability of nodes and allows a structured elicitation, and these causal Bayesian networks include the structure judgment of experts. Bayesian networks are also used in classifying students according to their performance, helping them to perform better. A Bayesian network with tree width is the property of the DAG used for building the network iteratively [38]. Identification of the parent set and an optimized structure allow easy learning in Bayesian networks. A dynamic Bayesian network deals with uncertainties following the addition of time-related information [39]. The probabilistic prediction model requires learning of the structure and parameters, as well as probabilistic reasoning. Causal relationships deal better with uncertainty, and they also act as a decision-making tool for determining the best strategies [40]. This is achieved using both qualitative and quantitative approaches, whereby the former involves structured learning for creating the DAG, and the latter uses the CPT of each node for calculation, as well as monitoring, diagnosing, and predicting justifiable and quantified decisions. Bayes’ theorem is best used as a predictor; therefore, higher bug prediction is also possible through a naïve Bayes classifier [41]. It also exhibits clinical decision-making, and it connects theory to evidence in clinical practice [42]. The alternate way of finding conditional probability is through Bayes’ theorem, which does not consider the joint probability [43]. It helps in mapping the problem onto an equation such as posterior probability, prior probability, likelihood, and evidence. Bayes’ theorem is explained very well in the analysis of cancer diagnostics. By using the values of sensitivity, specificity, base rate, and binary classification (which gives the true positive rate (TPR), also known as sensitivity, and the true negative rate (TNR), also known as specificity), and base rates for condition and prediction (probability of a positive class (PC), probability of a negative class (NC), probability of a positive prediction (PP)), the posterior probability known as precision can be found using the positive predictive value from the confusion matrix when the beliefs are known from the events. This theorem also gives the relationship between data and the model, which is calculated from the observed probabilities of the data given the hypothesis. The observed data reflect the probability of the hypothesis based on prior probability. In addition, if the observed probability P(D) increases, the probability of the hypothesis given the observed data P(h|D) decreases. On the other hand, if the probability of hypothesis P(h) and the probability of the observed data given hypothesis P(D|h) increase, the probability of the hypothesis given data P(h|D) also increases. Classification is framed by calculating the conditional probability of a class given the data.

This paper models Bayesian belief networks, where probabilistic models define the relationship between variables and are, thus, used for calculating probabilities [44]. These Bayesian networks are represented by a probabilistic graphical model, which finds the conditional dependence through directed edges. Through this Bayesian network, the relationships between nodes and edges are found given the evidence. Here, Bayesian probability represents the beliefs in an outcome by finding the joint probabilities of events, and it is used for making decisions. It considers a Bayesian network with 16 random variables, assuming conditional dependence in the presence of conditional independence.

## 3. Bayes Network Prediction for Telemedicine System

**Figure 1:**
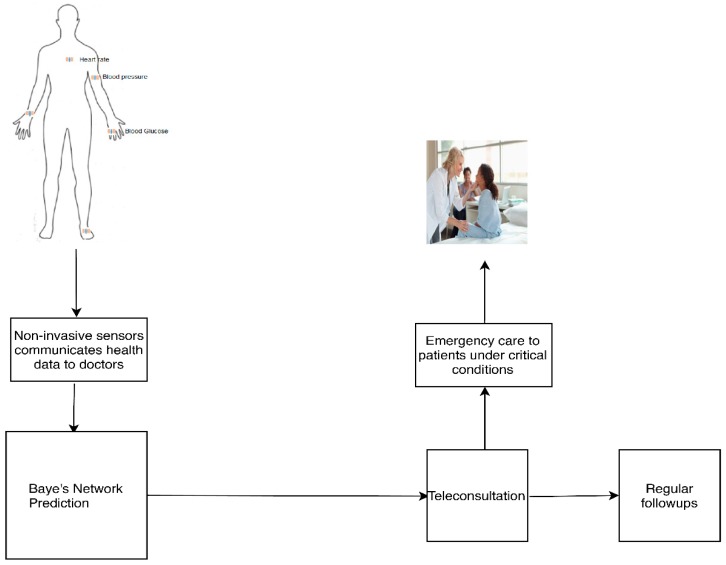
Schematic view of the methodology.

A patient wearing non-invasive sensors sends health data to remote healthcare centers for teleconsultation. The doctors examine the data at the healthcare centers and check whether the patient needs emergency care or teleconsultation through a Bayes network model for telemedicine. According to the output, teleconsultation or point of emergency care is given to the patient. Figure 1 shows the schematic representation of the telemedicine set-up.

**Figure 2:**
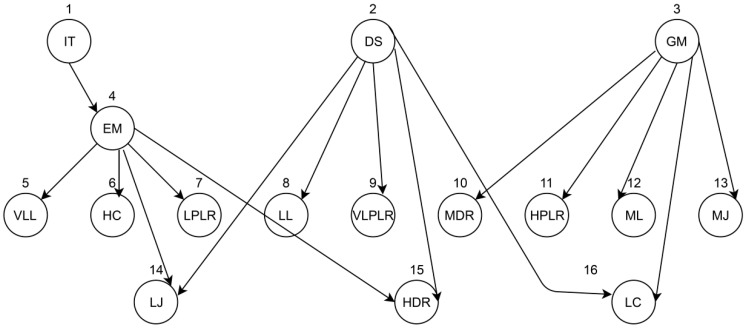
Bayes network model for telemedicine.

A Bayesian belief network model is formed from the conditions observed through a DAG, which represents the probabilistic relationships from events. The events are represented as immediate consultation, emergency monitoring, delay-sensitive monitoring, general monitoring, very low latency, low latency, medium latency, high cost, low cost, low jitter, medium jitter, very low packet loss rate, low packet loss rate, high packet loss rate, high data rate, and moderate data rate. The directed arrow represents the conditional probability between parent and child nodes, as shown in Figure 2. Firstly, the emergency condition event, which is the uncertainty situation, is possible when the conditions are very low latency (or) high cost (or) very low packet loss rate (or) high data rate (or) low jitter. Similarly, the delay-sensitive monitoring is the uncertainty situation when the conditions are low latency (or) moderate cost (or) low packet loss rate (or) low jitter. Similarly, the general monitoring is the uncertainty situation when the conditions are high latency (or) low cost (or) high packet loss rate (or) medium jitter. The purpose of the proposed system is to predict/determine the health of the patient with the help of the network parameters. This system is modeled for immediate teleconsultation, emergency monitoring, delay-sensitive monitoring, or general monitoring with the help of network parameters like latency, jitter, data rate, cost, and packet loss ratio. These nodes are connected according to the conditional dependence, as well as the causation between the nodes. The total number of nodes is 16 because, in this Bayes network, there are four network parameters to be analyzed for four possible monitoring conditions.

The probability values are assumed such that the emergency condition must be handled at the first priority. Second priority is given to delay-sensitive monitoring, and the least priority is given to general monitoring. Bayes’ theorem calculates the probability of occurrence in the future by incorporating prior knowledge. This method is considered as the best method because it learns from experience, and these predictions are stored in electronic records which can be used by a doctor when needed. The preferred explanation makes the evidence or observation more likely and, thus, the data fit the Bayes network model.

The proposed Bayes network model for telemedicine has 16 nodes, as shown in Figure 2. Each node has its CPT defined with probabilities, which also helps in finding conditional probability tables (CPTs) from the directed acyclic graph (DAG).

The Bayesian network is formed with nodes such as immediate teleconsultation (IT), emergency monitoring (EM), delay-sensitive monitoring (DS), and general monitoring (GM). IT occurs when there is need for EM. The EM condition is possible whenever there is very low latency (VLL), low packet loss rate (LPLR), low jitter (LJ), high cost (HC), and high data rate (HDR). The DS condition occurs when there is low jitter (LJ), high data rate (HDR), very low packet loss rate (VLPLR), low latency (LL), and low cost (LC). The GM condition is possible when there is low cost (LC), moderate data rate (MDR), high packet loss rate (HPLR), moderate latency (ML), and moderate jitter (MJ). If the network is the table of all possible combinations, it will be large. Thus, applying Bayesian nets only relates the nodes through causality, which greatly reduces the computation cost by finding the probabilities of related parent–child nodes. In addition, these are adaptable networks even with limited evidence, and they form new knowledge.

For this network, the values of probabilities in each node are derived by assuming 50 patients in a hospital, and the probabilities are assumed. The first node is the immediate teleconsultation node, and its parent node is in emergency monitoring. Its conditional probability is given by P(IT)=0.999. The second node is the delay node, and its parent node is in delay-sensitive monitoring. Its conditional probability is given by P(DS)=0.888. The third node is the general node, and its parent node is in general monitoring. Its conditional probability is given by P(GM)=0.777. The fourth node is emergency monitoring (EM). If IT is true, P(EM)=0.98. If IT is false P(~EM)=0.02. The fifth node is the very low latency (VLL) node, depending on EM. If EM is true, P(VLL)=0.90; otherwise, P(~VLL)=0.05.

The sixth node is the high cost (HC) node, depending on EM. If EM is true, P(HC)=0.70; otherwise, P(~HC)=0.30. The seventh node is the low packet loss rate (LPLR) node, depending on EM. If EM is true, P(LPLR)=0.95; otherwise, P(~LPLR)=0.05. The eighth node is the low latency (LL) node, depending on DS. If DS is true, P(LL)=0.94; otherwise, P(~LL)=0.06. The ninth node is the very low packet loss rate (VLPLR) node, depending on DS. If DS is true, P(VLPLR)=0.97. If DS is false, P(~VLPLR)=0.03. The 10th node is moderate data rate (MDR), depending on the GM. If GM is true, P(MDR)=0.99. If GM is false, P(~MDR)=0.01. The 11th node is high packet loss rate (HPLR). If GM is true, P(HPLR)=0.95. If GM is false, P(~HPLR)=0.05.

The 12th node is moderate latency (ML). If GM is true, P(ML)=0.96. If GM is false, P(~ML)=0.04. The 13th node is mixed jitter (MJ), depending on GM. If GM is true, P(MJ)=0.94. If GM is false, P(~MJ)=0.06. The 14th node is the low jitter (LJ) node, depending on EM and DS. If EM and DS are true, P(LJ)=0.95. If EM is true and DS is false, P(LJ)=0.94. If EM is false and DS is true, P(LJ)=0.74. If both EM and DS are false, P(LJ)=0.60. The 15th node is the high data rate (HDR), depending on EM and DS. If EM and DS are true, P(HDR)=0.96. If EM is true and DS is false, P(HDR)=0.92. If EM is false and DS is true, P(HDR)=0.74. If EM and DS are false P(HDR)=0.70. The 16th node is low cost (LC), depending on DS and GM. If both DS and GM are true, P(LC)=0.98. If DS is true and GM is false, P(LC)=0.94. If DS is false and GM is true, P(LC)=0.70. If DS and EM are true, P(LC)=0.60.

## 4. Bayes Network Prediction Model for Telemedicine

Each node is assumed to be conditionally independent of its immediate non-descendants, given by its immediate parents. Bayes’ theorem gives the compact representation of joint distribution. In the below DAG, there are 16 nodes. Thus, the computation of probabilities results in 2^16^ = 65,536 probabilities. However, through the CPT, there are only 28 conditional probabilities.

By analyzing the network model of Figure 2, there are three stages of nodes. The first-stage nodes include 1, 2, and 3, which deal with IT, DS, and GM. They have only two alternatives.

### 4.1. Probabilities of Nodes (1 to 3)

At node 1, the probability for finding emergency monitoring can be found when the network needs IT; then, P(IT)=0.999, and, when no IT is needed, P(~IT)=0.001.

At node 2, the probability for finding delay-sensitive monitoring can be found when the network is DS; then, P(DS)=0.888, and, when there is no DS, P(~DS)=0.112.

At node 3, the probability for finding general monitoring can be found when the network has GM; then, P(GM)=0.777, and, when it has no GM, P(~GM)=0.223.

### 4.2. Probabilities of Nodes (4 to 14)

The second-stage nodes include 4, 5, 6, 7, 8, 9, 10, 11, 12, 13, and 14, which have a hypothesis and degree of belief. They have pre-existing beliefs about their hypothesis and calculate their joint probabilities with the help of their priors and likelihood. Posterior probability can be found by using the prior probability and observed beliefs/evidence. It can be found from the updated probability of an event with new evidence given by Equation (1).

(1) $$P\left(A/B\right)=\frac{P\left(A\right)\cdot \;P(B/A)}{P\left(B\right)}.$$

At node 4, probabilistic reasoning can be applied with the set of candidate hypotheses with IT= immediate consultation and ~IT= no immediate teleconsultation, which is shown in Table 1 as $P\left(IT\right)=0.999=(p_{0})\;and\;P\left(\sim IT\right)=0.001=(p_{1})$, where $p_{0}+p_{1}=1$. If there are 50 patients in a hospital in an emergency condition and immediate consultation is needed, all patients are consulted immediately, which is given by the observed beliefs. The prior probabilities are $P\left(IT,EM\right)=\frac{49}{50}=0.98;\;P\left(IT,\sim EM\right)=\frac{2}{50}=0.04;\;P\left(\sim IT,\;EM\right)=\frac{1}{50}=0.02;\;P\left(\sim IT,\;\sim EM\right)=\frac{48}{50}=0.96$. From Equation (1), the posterior probabilities are given by Equation (A1) (Appendix A).

At node 5, probabilistic reasoning can be applied with the set of candidate hypotheses with EM = emergency condition and ~EM= no emergency condition, which is shown in Table 1 as $P\left(EM\right)=0.97904=(p_{0})\;and\;P\left(\sim EM\right)=0.04092=(p_{1})$, where $p_{0}+p_{1}=1$. The observed beliefs are revised, and, according to its prior probabilities,P(EM,VLL)=0.99768; P(EM,~VLL)=0.71578; P(~EM, VLL)=0.002316; P(~EM, ~VLL)=0.284212. From Equation (1), the posterior probabilities are given by Equation (A2) (Appendix A).

At node 6, probabilistic reasoning can be applied with the set of candidate hypotheses with EM = emergency condition and ~EM= no emergency condition, which is shown in Table 1 as $P\left(EM\right)=0.97904=(p_{0})\;and\;P\left(\sim EM\right)=0.002316=(p_{1}),$ where $p_{0}+p_{1}=1$. The observed beliefs are revised, and, according to prior probabilities, P(EM,HC)=0.98591; P(EM,~HC)=0.87879; P(~EM, HC)=0.000588; P(~EM, ~HC)=0.121209. From Equation (1), the posterior probabilities are given by Equation (A3) (Appendix A).

At node 7, probabilistic reasoning can be applied with the set of candidate hypotheses with EM = emergency condition and ~EM= no emergency condition, which is shown in Table 1 as $P\left(EM\right)=0.97904=(p_{0})\;and\;P\left(\sim EM\right)=0.04092=(p_{1})$, where $p_{0}+p_{1}=1$. The observed beliefs are revised, and, according to prior probabilities, P(EM,LPLR)=0.998248; P(EM,~LPLR)=0.554791; P(~EM, LPLR)=0.0017567; P(~EM, ~LPLR)=0.44521. From Equation (1), the posterior probabilities are given by Equation (A4) (Appendix A).

At node 8, probabilistic reasoning can be applied with the set of candidate hypotheses with DS = delay-sensitive monitoring and ~DS= no delay-sensitive monitoring, as shown in Table 1 as $P\left(DS\right)=0.888=(p_{0})\;and\;P\left(\sim DS\right)=0.112=(p_{1})$, where $p_{0}+p_{1}=1$. The observed beliefs are revised, and, according to prior probabilities, P(DS,LL)=0.992013; P(DS,~LL)=0.408088; P(~DS, LL)=0.007986; P(~DS, ~LL)=0.591911. From Equation (1), the posterior probabilities are given by Equation (A5) (Appendix A).

At node 9, probabilistic reasoning can be applied with the set of candidate hypotheses with DS = delay-sensitive monitoring and ~DS= no delay-sensitive monitoring, as shown in Table 1 as $P\left(DS\right)=0.888=(p_{0})\;and\;P\left(\sim DS\right)=0.223=(p_{1})$, where $p_{0}+p_{1}=1$. The observed beliefs are revised, and, according to prior probabilities, P(DS,VLPLR)=0.98938; P(DS,~VLPLR)=0.37121; P(~DS, VLPLR)=0.003885; P(~DS, ~VLPLR)=0.62626. From Equation (1), the posterior probabilities are given by Equation (A6) (Appendix A).

At node 10, probabilistic reasoning can be applied with the set of candidate hypotheses with GM = general monitoring and ~GM= no general monitoring, as shown in Table 1 as $P\left(GM\right)=0.777=(p_{0})\;and\;P\left(\sim GM\right)=0.223=(p_{1})$, where $p_{0}+p_{1}=1$. The observed beliefs are revised, and, according to prior probabilities, P(GM,MDR)=0.997109; P(GM,~MDR)=0.066387; P(~GM,MDR)=0.0028906; P(~GM, ~MDR)=0.933612. From Equation (1), the posterior probabilities are given by Equation (A7) (Appendix A).

At node 11, probabilistic reasoning can be applied with the set of candidate hypotheses with GM = general monitoring and ~GM= no general monitoring, as shown in Table 1 as $P\left(GM\right)=0.777=(p_{0})\;and\;P\left(\sim GM\right)=0.223=(p_{1})$, where $p_{0}+p_{1}=1$. The observed beliefs are revised, and, according to prior probabilities,P(GM,HPLR)=0.98511; P(GM,~HPLR)=0.09727; P(~GM,HPLR)=0.014880; P(~GM, ~HPLR)=0.90272. From Equation (1), the posterior probabilities are given by Equation (A8) (Appendix A).

At node 12, probabilistic reasoning can be applied with the set of candidate hypotheses with GM = general monitoring and ~GM= no general monitoring, as shown in Table 1 as $P\left(GM\right)=0.777=(p_{0})\;and\;P\left(\sim GM\right)=0.223=(p_{1})$, where $p_{0}+p_{1}=1$. The observed beliefs are revised, and, according to prior probabilities, P(GM,ML)=0.98857; P(GM,~ML)=0.033998; P(~GM,ML)=0.01182; P(~GM, ~ML)=0.96600. From Equation (1), the posterior probabilities are given by Equation (A9) (Appendix A).

At node 13, probabilistic reasoning can be applied with the set of candidate hypotheses with GM = general monitoring and ~*GM* = no general monitoring, as shown in Table 1 as $P\left(GM\right)=0.777=(p_{0})\;and\;P\left(\sim GM\right)=0.223=(p_{1})$, where $p_{0}+p_{1}=1$. The observed beliefs are revised, and, according to prior probabilities, P(GM,MJ)=0.98201; P(GM,~MJ)=0.15496; P(~GM,MJ)=0.017989; P(~GM, ~MJ)=0.84503. From Equation (1), the posterior probabilities are given by Equation (A10) (Appendix A).

The third-stage nodes include 14, 15, and 16, which depend on the hypotheses of two nodes and the degree of belief. They have pre-existing beliefs about their hypothesis, and they calculate their joint probabilities with the help of their priors and likelihood.

At node 14, probabilistic reasoning can be applied with the following set of candidate hypotheses:

Case 1: If EM,DS are true, P(LJ)=0.95, P(~LJ)=0.05;

Case 2: If EM is true, DS is false, P(LJ)=0.94, P(~LJ)=0.06;

Case 3: If EM is false, DS is true, P(LJ)=0.74, P(~LJ)=0.26;

Case 4: If EM,DS are false, P(LJ)=0.60, P(~LJ)=0.40.

The observed beliefs are revised as follows according to prior probabilities:

$$\begin{array}{c} P\left(EM,DS\;|LJ\right)=0.7;\;P\left(EM,DS|\;\sim LJ\right)=0.3;\\ P\left(EM,\sim DS\;|LJ\right)=0.6;\;P\left(\sim EM,DS|\;\sim LJ\right)=0.4;\\ P\left(\sim EM,DS\;|LJ\right)=0.5;\;P\left(\sim EM,DS|\;\sim LJ\right)=0.5;\\ P\left(\sim EM,\sim DS|LJ\right)=0.8;P\left(\sim EM,\sim DS|\sim LJ\right)=0.2. \end{array}$$

From Equation (1), the posterior probabilities are given by Equation (A11) (Appendix A).

At node 15, probabilistic reasoning can be applied with the following set of candidate hypotheses:

Case 1: If EM,DS are true, P(HDR)=0.96, P(~HDR)=0.04;

Case 2: If EM is true, DS is false, P(HDR)=0.92, P(~HDR)=0.08;

Case 3: If EM is false, DS is true, P(HDR)=0.74, P(~HDR)=0.26;

Case 4: If EM,DS are false, P(HDR)=0.70, P(~HDR)=0.30.

The observed beliefs are revised as follows according to prior probabilities:

P(EM,DS |HDR)=0.6; P(EM,DS| ~HDR)=0.4;P(EM,~DS |HDR)=0.5; P(~EM,DS| ~HDR)=0.5;P(~EM,DS |HDR)=0.7; P(~EM,DS| ~HDR)=0.3;P(~EM,~DS|HDR)=0.8;P(~EM,~DS|~HDR)=0.2.

From Equation (1), the posterior probabilities are given by Equation (A12) (Appendix A).

At node 16, probabilistic reasoning can be applied with the following set of candidate hypotheses:

Case 1: If DS,GM are true, P(LC)=0.98, P(~LC)=0.02;

Case 2: If *DS is true*, *GM is false*, *P*(*LC*) = 0.94, *P*(~*LC*) = 0.06;

Case 3: If DS is false, GM is true, P(LC)=0.70, P(~LC)=0.30;

Case 4: If DS,GM are false, P(LC)=0.60, P(~LC)=0.40.

The observed beliefs are revised as follows according to prior probabilities:

$$\begin{array}{c} P\left(DS,GM\;|LC\right)=0.7;\;P\left(DS,GM|\;\sim LC\right)=0.3;\\ P\left(DS,\sim GM\;|LC\right)=0.6;\;P\left(DS,\sim GM|\;\sim LC\right)=0.4;\\ P\left(\sim DS,GM\;|LC\right)=0.5;\;P\left(\sim DS,GM|\;\sim LC\right)=0.5;\\ P\left(\sim DS,\sim GM|LC\right)=0.8;P\left(\sim DS,\sim GM|\sim LC\right)=0.2. \end{array}$$

From Equation (1), the posterior probabilities are given by Equation (A13) (Appendix A).

## 5. Results and Discussion

Bayes theorem deceptively calculates the conditional probabilities used for developing classification models. The scenario for Bayes network prediction is that a patient in a hospital may or may not need immediate consultation (immediate consultation is true or false) and a doctor determines whether the patient is having an emergency condition or not. The problem statement is as follows: if a patient is randomly selected by a doctor for checking emergency condition, the probability is that the patient needs immediate consultation.

### Manual Calculation

In this scenario, sometimes the patient needs immediate consultation, but the doctor may not determine whether the patient is in an emergency condition. The patient’s situation is that they may need immediate consultation, which can be found through the sensitivity (true positive rate). In this case, the assumption of probability of immediate consultation is high, i.e., 49 persons in 50 cases are consulted immediately, given by P(IT = true) = 0.999 and the prior probability of a patient found through the observed beliefs. Thus, the probability of a patient who needs immediate consultation is given priority, and the doctor selects the patient who is in emergency condition, which can be found using Bayes’ theorem [43]. Thus, the posterior probability can be found as follows

- P(A/B) = P(B/A) × P(B);
- As assumed through CPT, posterior probability P(IT|EM) = (P(EM|IT) × P(IT))/P(EM);
- (P(EM|IT) = 0.98 and P(IT) = 0.999;
- P(IT|EM) = (0.98 × 0.999)/P(EM);
- P(B) can be found using P(B) = P(B|A) × P(A) + P(B|~A) × P(~A);
- P(EM) = P(EM|IT) × P(IT) + P(EM|~IT) × P(~IT);
- P(EM) = 0.98 × 0.999 + 0.02 × 0.001 = 0.97904;
- Finally, the posterior probability is found using P(IT|EM) = (0.98 × 0.999)/0.97904 = 1.000.

Similarly, every posterior probability of all the events in the DAG is found and appended in Appendix A. In the given scenario, three pieces of information are needed, i.e., prior probability, likelihood (sensitivity or true positive rate), and evidence (specificity or true negative rate). Thus, the confusion matrix can be defined as shown in Table 2.

From the confusion matrix shown in Table 1, the following statements apply:

- P(B|A) = sensitivity, true positive rate (TPR) = TP/(TP + FN) = P(EM|IT) = 0.98;
- P(B|~A) = false positive rate (FPR) = FP/(FP + TN) = P(EM|~IT) = 0.02;
- P(~B|~A) = specificity, true negative rate (TNR) = TN/(TN + FP) = P(~EM|~IT) = 0.96;
- P(~B|A) = false negative rate (FNR) = FN/(FN + TP) = P(~EM|IT = T) = 0.04.

By mapping the prior probabilities for the emergency condition (class) and the immediate consultation (prediction), the following statements apply:

- P(A) = probability of a positive class (PC) = P(IT) = 0.999;
- P(~A) = probability of a negative class (NC) = P(~IT) = 0.001;
- P(B) = probability of a positive prediction (PP) = P(EM) = 0.97904;
- P(~B) = probability of a negative prediction (NP) = P(~EM) = 0.02096.

Thus, Bayes’ theorem can be written as follows:

- P(A|B) = (TPR × PC)/PP = (P(EM|IT) × P(IT))/P(EM);
- P(B) = TPR × PC + FPR × NC = P(EM|IT) × P(IT) + P(EM|~IT) × P(~IT).

The posterior probabilities calculated using Bayes’ theorem is the precision, known as the positive predictive value (PPV), computed from confusion matrix.

- PPV = TP/(TP + FP);
- P(A|B) = PPV = TPR × PC/PP.

Finally, the prediction is given as follows:

- P(IT|EM) = P(EM|IT) × P(IT)/P(EM);
- P(IT|EM) = 0.98 × 0.999/0.97904 = 0.999979.

Likewise, the probabilities can be found for delay-sensitive monitoring and general monitoring cases also. Using this method, probabilities of any number of patient cases can be easily calculated and evaluated. By knowing the Bayesian belief network model, from the conditional probability tables, the probabilities of uncertain conditions can be determined with this method. The results are posterior probabilities calculated from the hypothesis and degree of belief in each node, and they are tabulated in Table 3 and in Table 4.

## 6. Conclusions

Recent WBAN papers applying telemedicine for emergency were collected and briefly presented. In addition, Bayes’ network-based prediction for emergency condition was analyzed, and its posterior probabilities were found. Bayes thinking can be applied when there is uncertainty in data. The inputs are linked to the output through probability levels, and these help in decision-making. Here, this technique was applied for decision-making with respect to immediate teleconsultation. As there is no confusion matrix for a population of people with immediate consultation and one without immediate consultation, which have emergency conditions and do not have emergency conditions, this method is easily applicable. Instead, prior probabilities with regard to the population and emergency condition are only known. This is suitable for beliefs that are known and when calculation is not possible in the real world. This helps in determining the probability of sending emergency messages to a remote doctor and determining how fast emergency messages can be sent. It can be observed that machine learning applies Bayes’ theorem for classifying predictive things. This method of testing any number of models on a dataset is done by finding the probability of each hypothesis with the true given data. In addition, maximum a posteriori (MAP) estimation for linear regression can be applied for prediction, and binary classification can be used as an alternative for maximum likelihood estimation (MLE). These networks are also applied in monitoring health outcomes and analysis.

## Figures and Tables

**Table 1:** Conditional probability table (CPT) for each node in the directed acyclic graph (DAG). IT—immediate teleconsultation; DS—delay-sensitive monitoring; GM—general monitoring; EM—emergency monitoring; VLL—very low latency; HC—high cost; LPLR—low packet loss rate; LL—low latency; VLPLR—very low packet loss rate; MDR—moderate data rate; HPLR—high packet loss rate; ML—moderate latency; MJ—moderate jitter; LJ—low jitter; HDR—high data rate; LC—low cost.

Node No.	Conditions	Probabilities
Node 1	If IT is true	P(IT) = 0.999
Node 2	If DS is true	P(DS) = 0.888
Node 3	If GM is true	P(GM) = 0.777
Node 4	If IT is true	P(EM) = 0.98
	If IT is false	P(~EM) = 0.02
Node 5	If EM is true	P(VLL) = 0.90
	If EM is false	P(~VLL) = 0.05
Node 6	If EM is true	P(HC) = 0.70
	If EM is false	P(~HC) = 0.30
Node 7	If EM is true	P(LPLR) = 0.95
	If EM is false	P(~LPLR) = 0.05
Node 8	If DS is true	P(LL) = 0.94
	If DS is false	P(~LL) = 0.06
Node 9	If DS is true	P(VLPLR) = 0.97
	If DS is false	P(~VLPLR) = 0.03
Node 10	If GM is true	P(MDR) = 0.99
	If GM is false	P(~MDR) = 0.01
Node 11	If GM is true	P(HPLR) = 0.95
	If GM is false	P(~HPLR) = 0.05
Node 12	If GM is true	P(ML) = 0.96
	If GM is false	P(~ML) = 0.04
Node 13	If GM is true	P(MJ) = 0.94
	If GM is false	P(~MJ) = 0.06
Node 14	If EM and DS are true	P(LJ) = 0.95; P(~LJ) = 0.05
	If EM is true and DS is false	P(LJ) = 0.94; P(~LJ) = 0.06
	If EM is false and DS is true	P(LJ) = 0.74; P(~LJ) = 0.26
	If EM and DS are false	P(LJ) = 0.60; P(LJ) = 0.40
Node 15	If EM and DS are true	P(HDR) = 0.96; P(~HDR) = 0.04
	If EM is true and DS is false	P(HDR) = 0.92; P(~HDR) = 0.08
	If EM is false and DS is true	P(HDR) = 0.74; P(~HDR) = 0.26
	If EM and DS are false	P(HDR) = 0.70; P(~HDR) = 0.30
Node 16	If DS and GM are true	P(LC) = 0.98; P(~LC) = 0.02
	If DS is true and GM is false	P(LC) = 0.94; P(~LC) = 0.06
	If DS is false and GM is true	P(LC) = 0.70; P(~LC) = 0.30
	If DS and GM are false	P(LC) = 0.60; P(~LC) = 0.40

**Table 2:** Confusion matrix.

	Positive Class
Positive prediction	True positive (TP)
Negative prediction	False negative (FN)

**Table 3:** Posterior probabilities for nodes (4–13).

Node No.	Conditions	Posterior Probabilities
Node 4	P(EM) = 0.97904 P(~EM) = 0.04092	P(IT|EM) = 0.999979 P(IT|~EM) = 0.976539 P(~IT|EM) = 0.00002 P(~IT|~EM) = 0.02346
Node 5	P(VLL) = 0.883182 P(~VLL) = 0.136778	P(EM|VLL) = 0.99768 P(EM|~VLL) = 0.71578 P(~EM|VLL) = 0.002316 P(~EM|~VLL) = 0.284212
Node 6	P(HC) = 0.695118 P(~HC) = 0.334222	P(EM|HC) =0.98591 P(EM|~HC) = 0.87879 P(~EM|HC) = 0.000588 P(~EM|~HC) = 0.121209
Node 7	P(LPLR) = 0.93172 P(~LPLR) = 0.088235	P(EM| LPLR) = 0.998248 P(EM|~ LPLR) = 0.554791 P(~EM| LPLR) = 0.0017567 P(~EM|~ LPLR) = 0.44521
Node 8	P(LL) = 0.84144 P(~LL) = 0.17408	P(DS|LL) = 0.992013 P(DS|~LL) = 0.408088 P(~DS|LL) = 0.007986 P(~DS|~LL) = 0.591911
Node 9	P(VLPLR) = 0.86472 P(~VLPLR) = 0.16632	P(DS|VLPLR) = 0.98938 P(DS|~VLPLR) = 0.37121 P(~DS|VLPLR) = 0.003885 P(~DS|~VLPLR) = 0.62626
Node 10	P(MDR) = 0.77146 P(~MDR) = 0.23408	P(GM|MDR) = 0.997109 P(GM|~MDR) = 0.066387 P(~GM|MDR) = 0.0028906 P(~GM|~MDR) = 0.933612
Node 11	P(HPLR) = 0.7493 P(~HPLR) = 0.23962	P(GM|HPLR) = 0.98511 P(GM|~HPLR) = 0.09727 P(~GM|HPLR) = 0.014880 P(~GM|~HPLR) = 0.90272
Node 12	P(ML) = 0.75454 P(~ML) = 0.22854	P(GM|ML) = 0.98857 P(GM|~ML) = 0.033998 P(~GM|ML) = 0.01182 P(~GM|~ML) = 0.96600
Node 13	P(MJ) = 0.74376 P(~MJ) = 0.2507	P(GM|MJ) = 0.98201 P(GM|~MJ) = 0.15496 P(~GM|MJ) = 0.017989 P(~GM|~MJ) = 0.84503

**Table 4:** Posterior probabilities for nodes (14–16).

Node No.	Conditions	Posterior Probabilities
Node 14	If EM is true and DS is true and {P(LJ) = 0.95 and P(~LJ) = 0.05} If EM is true and DS is false and {P(LJ) = 0.94 and P(~LJ) = 0.06} If EM is false and DS is true and {P(LJ) = 0.74 and P(~LJ) = 0.26} If EM is false and DS is false and {P(LJ) = 0.60 and P(~LJ) = 0.40}	P(EM, DS|LJ) = 0.380 and P(EM, DS|~LJ) = 0.163 P(EM, ~DS|LJ) = 0.130 and P(EM, ~DS|~LJ) = 0.087 P(~EM, DS|LJ) = 0.108 and P(~EM, DS|~LJ) = 0.108 P(~EM, ~DS|LJ) = 0.087and P(~EM, ~DS|~LJ) = 0.222
Node 15	If EM is true and DS is true and {P(HDR) = 0.96 and P(~HDR) = 0.04} If EM is true and DS is false and {P(HDR) = 0.92 and P(~HDR) = 0.08} If EM is true and DS is true and {P(HDR) = 0.74 and P(~HDR) = 0.26} If EM is true and DS is false and {P(HDR) = 0.70 and P(~HDR) = 0.30}	P(EM, DS|HDR) = 0.525 and P(EM, DS|~HDR) = 0.35 P(EM, ~DS|HDR) = 0.0625 and P(EM, ~DS|~HDR) = 0.0625 P(~EM, DS|HDR) = 0.0875 and P(~EM, DS|~HDR) = 0.0375 P(~EM, ~DS|HDR) = 0.1 and P(~EM, ~DS|~HDR) = 0.025
Node 16	If DS is true and GM is true and {P(LC) = 0.98 and P(~LC) = 0.02} If DS is true and GM is false and {P(LC) = 0.94 and P(~LC) = 0.06} If DS is true and GM is true and {P(LC) = 0.70 and P(~LC) = 0.30} If EM is true and GM is false and {P(LC) = 0.60 and P(~LC) = 0.40}	P(DS, GM|LC) = 0.42 and P(DS, GM|~LC) = 0.18 P(DS, ~GM|LC) = 0.12 and P(DS, ~GM|~LC) = 0.08 P(~DS, GM|LC) = 0.05 and P(~DS, GM|~LC) = 0.05 P(~DS, ~GM|LC) = 0.08 and P(~DS, ~GM|~LC) = 0.02

## Appendix A

This appendix contains the calculation of posterior probability details derived from the observed beliefs in the conditional probability tables.

(A1) $$P\left(IT|EM\right)=\frac{P\left(EM,IT\right)P\left(IT\right)}{P\left(EM\right)}=\frac{0.98\times 0.999}{0.97904}=0.999979,$$

$$P\left(IT|\sim EM\right)=\frac{P\left(\sim EM,IT\right)P\left(IT\right)}{P\left(\sim EM\right)}=\frac{0.04\times 0.999}{0.04092}=0.976539,$$

$$P\left(\sim IT|EM\right)=\frac{P\left(EM,\sim IT\right)P\left(\sim IT\right)}{P\left(EM\right)}=\frac{0.02\times 0.001}{0.97904}=0.00002,$$

$$P\left(\sim IT|\sim EM\right)=\frac{P\left(\sim EM,\sim IT\right)P\left(\sim IT\right)}{P\left(\sim EM\right)}=\frac{0.96\times 0.001}{0.04092}=0.02346.$$

(A2) $$P\left(EM|VLL\right)=\frac{P\left(VLL,EM\right)P\left(EM\right)}{P\left(VLL\right)}=\frac{0.90\times 0.97904}{0.883182}=0.99768,$$

$$P\left(EM|\sim VLL\right)=\frac{P\left(\sim VLL,\;EM\right)P\left(EM\right)}{P\left(\sim VLL\right)}=\frac{0.10\times 0.97904}{0.136778}=0.71578,$$

$$P\left(\sim EM|VLL\right)=\frac{P\left(\;VLL,\sim EM\right)P\left(\sim EM\right)}{P\left(VLL\right)}=\frac{0.05\times 0.04092}{0.883182}=0.002316,$$

$$P\left(\sim EM|\sim VLL\right)=\frac{P\left(\sim VLL,\;\sim EM\right)P\left(\sim EM\right)}{P\left(\sim VLL\right)}=\frac{0.95\times 0.04092}{0.136778}=0.284212.$$

(A3) $$P\left(EM|HC\right)=\frac{P\left(HC,EM\right)P\left(EM\right)}{P\left(HC\right)}=\frac{0.70\times 0.97904}{0.695118}=0.98591,$$

$$P\left(EM|\sim HC\right)=\frac{P\left(\sim HC,\;EM\right)P\left(EM\right)}{P\left(\sim HC\right)}=\frac{0.30\times 0.97904}{0.334222}=0.87879,$$

$$P\left(\sim EM|HC\right)=\frac{P\left(HC,\sim EM\right)P\left(\sim EM\right)}{P\left(HC\right)}=\frac{0.01\times 0.04092}{0.695118}=0.000588,$$

$$P\left(\sim EM|\sim HC\right)=\frac{P\left(\sim HC,\;\sim EM\right)P\left(\sim EM\right)}{P\left(\sim HC\right)}=\frac{0.99\times 0.04092}{0.334222}=0.121209.$$

(A4) $$P\left(EM|LPLR\right)=\frac{P\left(LPLR,EM\right)P\left(EM\right)}{P\left(LPLR\right)}=\frac{0.95\times 0.97904}{0.93172}=0.998248,$$

$$P\left(EM|\sim LPLR\right)=\frac{P\left(\sim LPLR,\;EM\right)P\left(EM\right)}{P\left(\sim LPLR\right)}=\frac{0.05\times 0.97904}{0.088235}=0.554791,$$

$$P\left(\sim EM|LPLR\right)=\frac{P\left(LPLR,\sim EM\right)P\left(\sim EM\right)}{P\left(LPLR\right)}=\frac{0.04\times 0.04092}{0.93172}=0.0017567,$$

$$P\left(\sim EM|\sim LPLR\right)=\frac{P\left(\sim LPLR,\;\sim EM\right)P\left(\sim EM\right)}{P\left(\sim LPLR\right)}=\frac{0.96\times 0.04092}{0.088235}=0.44521.$$

(A5) $$P\left(DS|LL\right)=\frac{P\left(LL,DS\right)P\left(DS\right)}{P\left(LL\right)}=\frac{0.94\times 0.888}{0.84144}=0.992013,$$

$$P\left(DS|\sim LL\right)=\frac{P\left(\sim LL,\;DS\right)P\left(DS\right)}{P\left(\sim LL\right)}=\frac{0.08\times 0.888}{0.17408}=0.408088,$$

$$P\left(\sim DS|LL\right)=\frac{P\left(LL,\sim DS\right)P\left(\sim DS\right)}{P\left(LL\right)}=\frac{0.06\times 0.112}{0.84144}=0.007986,$$

$$P\left(\sim DS|\sim LL\right)=\frac{P\left(\sim LL,\;\sim DS\right)P\left(\sim DS\right)}{P\left(\sim LL\right)}=\frac{0.92\times 0.112}{0.17408}=0.591911.$$

(A6) $$P\left(DS|VLPLR\right)=\frac{P\left(VLPLR,DS\right)P\left(DS\right)}{P\left(VLPLR\right)}=\frac{0.97\times 0.882}{0.86472}=0.98938,$$

$$P\left(DS|\sim VLPLR\right)=\frac{P\left(\sim VLPLR,\;DS\right)P\left(DS\right)}{P\left(\sim VLPLR\right)}=\frac{0.07\times 0.882}{0.16632}=0.37121,$$

$$P\left(\sim DS|VLPLR\right)=\frac{P\left(VLPLR,\sim DS\right)P\left(\sim DS\right)}{P\left(VLPLR\right)}=\frac{0.03\times 0.112}{0.86472}=0.003885,$$

$$P\left(\sim DS|\sim VLPLR\right)=\frac{P\left(\sim VLPLR,\;\sim DS\right)P\left(\sim DS\right)}{P\left(\sim VLPLR\right)}=\frac{0.93\times 0.112}{0.16632}=0.62626.$$

(A7) $$P\left(GM|MDR\right)=\frac{P\left(MDR,GM\right)P\left(GM\right)}{P\left(MDR\right)}=\frac{0.99\times 0.777}{0.77146}=0.997109,$$

$$P\left(GM|\sim MDR\right)=\frac{P\left(\sim MDR,\;GM\right)P\left(GM\right)}{P\left(\sim MDR\right)}=\frac{0.02\times 0.777}{0.23408}=0.066387,$$

$$P\left(\sim GM|MDR\right)=\frac{P\left(MDR,\sim GM\right)P\left(\sim GM\right)}{P\left(MDR\right)}=\frac{0.01\times 0.223}{0.77146}=0.0028906,$$

$$P\left(\sim GM|\sim MDR\right)=\frac{P\left(\sim MDR,\;\sim GM\right)P\left(\sim GM\right)}{P\left(\sim MDR\right)}=\frac{0.98\times 0.223}{0.23408}=0.933612.$$

(A8) $$P\left(GM|HPLR\right)=\frac{P\left(HPLR,GM\right)P\left(GM\right)}{P\left(HPLR\right)}=\frac{0.95\times 0.777}{0.7493}=0.98511,$$

$$P\left(GM|\sim HPLR\right)=\frac{P\left(\sim HPLR,\;GM\right)P\left(GM\right)}{P\left(\sim HPLR\right)}=\frac{0.03\times 0.777}{0.23962}=0.09727,$$

$$P\left(\sim GM|HPLR\right)=\frac{P\left(HPLR,\sim GM\right)P\left(\sim GM\right)}{P\left(HPLR\right)}=\frac{0.05\times 0.223}{0.7493}=0.014880,$$

$$P\left(\sim GM|\sim HPLR\right)=\frac{P\left(\sim HPLR,\;\sim GM\right)P\left(\sim GM\right)}{P\left(\sim HPLR\right)}=\frac{0.97\times 0.223}{0.23962}=0.90272.$$

(A9) $$P\left(GM|ML\right)=\frac{P\left(ML,GM\right)P\left(GM\right)}{P\left(ML\right)}=\frac{0.96\times 0.777}{0.75454}=0.98857,$$

$$P\left(GM|\sim ML\right)=\frac{P\left(\sim ML,\;GM\right)P\left(GM\right)}{P\left(\sim ML\right)}=\frac{0.01\times 0.777}{0.22854}=0.033998,$$

$$P\left(\sim GM|ML\right)=\frac{P\left(ML,\sim GM\right)P\left(\sim GM\right)}{P\left(ML\right)}=\frac{0.04\times 0.223}{0.75454}=0.01182,$$

$$P\left(\sim GM|\sim ML\right)=\frac{P\left(\sim ML,\;\sim GM\right)P\left(\sim GM\right)}{P\left(\sim ML\right)}=\frac{0.99\times 0.223}{0.22854}=0.96600.$$

(A10) $$P\left(GM|MJ\right)=\frac{P\left(MJ,GM\right)P\left(GM\right)}{P\left(MJ\right)}=\frac{0.94\times 0.777}{0.74376}=0.98201,$$

$$P\left(GM|\sim MJ\right)=\frac{P\left(\sim MJ,\;GM\right)P\left(GM\right)}{P\left(\sim MJ\right)}=\frac{0.05\times 0.777}{0.2507}=0.15496,$$

$$P\left(\sim GM|MJ\right)=\frac{P\left(MJ,\sim GM\right)P\left(\sim GM\right)}{P\left(MJ\right)}=\frac{0.06\times 0.223}{0.74376}=0.017989,$$

$$P\left(\sim GM|\sim MJ\right)=\frac{P\left(\sim MJ,\;\sim GM\right)P\left(\sim GM\right)}{P\left(\sim MJ\right)}=\frac{0.95\times 0.223}{0.2507}=0.84503.$$

(A11) $$P\left(EM,DS|LJ\right)=\frac{P\left(LJ|EM,DS\right)P\left(EM,DS\right)}{P\left(LJ\right)}=\frac{0.7\times 0.3}{0.92}=0.38,$$

$$P\left(EM,\sim DS|LJ\right)=\frac{P\left(LJ|EM,\sim DS\right)P\left(EM,\sim DS\right)}{P\left(LJ\right)}=\frac{0.6\times 0.2}{0.92}=0.13,$$

$$P\left(\sim EM,DS|LJ\right)=\frac{P\left(LJ|\sim EM,DS\right)P\left(\sim EM,DS\right)}{P\left(LJ\right)}=\frac{0.5\times 0.2}{0.92}=0.108,$$

$$P\left(\sim EM,\sim DS|LJ\right)=\frac{P\left(LJ|\sim EM,\sim DS\right)P\left(\sim EM,\sim DS\right)}{P\left(LJ\right)}=\frac{0.8\times 0.1}{0.92}=0.087,$$

$$P\left(EM,DS|\sim LJ\right)=\frac{P\left(\sim LJ|EM,DS\right)P\left(EM,DS\right)}{P\left(\sim LJ\right)}=\frac{0.3\times 0.5}{0.92}=0.163,$$

$$P\left(EM,\sim DS|\sim LJ\right)=\frac{P\left(\sim LJ|EM,\sim DS\right)P\left(EM,\sim DS\right)}{P\left(\sim LJ\right)}=\frac{0.4\times 0.2}{0.92}=0.087,$$

$$P\left(\sim EM,DS|\sim LJ\right)=\frac{P\left(\sim LJ|\sim EM,DS\right)P\left(\sim EM,DS\right)}{P\left(\sim LJ\right)}=\frac{0.5\times 0.2}{0.92}=0.108,$$

$$P\left(\sim EM,\sim DS|\sim LJ\right)=\frac{P\left(LJ|\sim EM,\sim DS\right)P\left(\sim EM,\sim DS\right)}{P\left(\sim LJ\right)}=\frac{0.2\times 0.1}{0.92}=0.022.$$

(A12) $$P\left(EM,DS|HDR\right)=\frac{P\left(HDR|EM,DS\right)P\left(EM,DS\right)}{P\left(HDR\right)}=\frac{0.6\times 0.7}{0.8}=0.525,$$

$$P\left(EM,\sim DS|HDR\right)=\frac{P\left(HDR|EM,\sim DS\right)P\left(EM,\sim DS\right)}{P\left(HDR\right)}=\frac{0.5\times 0.1}{0.8}=0.0625,$$

$$P\left(\sim EM,DS|HDR\right)=\frac{P\left(HDR|\sim EM,DS\right)P\left(\sim EM,DS\right)}{P\left(HDR\right)}=\frac{0.7\times 0.1}{0.8}=0.0875,$$

$$P\left(\sim EM,\sim DS|HDR\right)=\frac{P\left(HDR|\sim EM,\sim DS\right)P\left(\sim EM,\sim DS\right)}{P\left(HDR\right)}=\frac{0.8\times 0.1}{0.8}=0.1,$$

$$P\left(EM,DS|\sim HDR\right)=\frac{P\left(\sim HDR|EM,DS\right)P\left(EM,DS\right)}{P\left(\sim HDR\right)}=\frac{0.4\times 0.7}{0.8}=0.35,$$

$$P\left(EM,\sim DS|\sim HDR\right)=\frac{P\left(\sim HDR|EM,\sim DS\right)P\left(EM,\sim DS\right)}{P\left(\sim HDR\right)}=\frac{0.5\times 0.1}{0.8}=0.0625,$$

$$P\left(\sim EM,DS|\sim HDR\right)=\frac{P\left(\sim HDR|\sim EM,DS\right)P\left(\sim EM,DS\right)}{P\left(\sim HDR\right)}=\frac{0.3\times 0.1}{0.8}=0.0375,$$

$$P\left(\sim EM,\sim DS|\sim HDR\right)=\frac{P\left(HDR|\sim EM,\sim DS\right)P\left(\sim EM,\sim DS\right)}{P\left(\sim HDR\right)}=\frac{0.2\times 0.1}{0.8}=0.025.$$

(A13) $$P\left(DS,GM|LC\right)=\frac{P\left(LC|DS,GM\right)P\left(DS,GM\right)}{P\left(LC\right)}=\frac{0.7\times 0.6}{0.1}=0.42,$$

$$P\left(DS,\sim GM|LC\right)=\frac{P\left(LC|DS,\sim GM\right)P\left(DS,\sim GM\right)}{P\left(LC\right)}=\frac{0.6\times 0.2}{0.1}=0.12,$$

$$P\left(\sim DS,GM|LC\right)=\frac{P\left(LC|\sim DS,\;GM\right)P\left(\sim DS,GM\right)}{P\left(LC\right)}=\frac{0.5\times 0.1}{0.1}=0.05,$$

$$P\left(\sim DS,\sim GM|LC\right)=\frac{P\left(LC|\sim DS,\sim GM\right)P\left(\sim DS,\sim GM\right)}{P\left(LC\right)}=\frac{0.8\times 0.1}{0.1}=0.08,$$

$$P\left(DS,GM|\sim LC\right)=\frac{P\left(\sim LC|DS,GM\right)P\left(DS,GM\right)}{P\left(\sim LC\right)}=\frac{0.3\times 0.6}{0.1}=0.18,$$

$$P\left(DS,\sim GM|\sim LC\right)=\frac{P\left(\sim LC|DS,\sim GM\right)P\left(DS,\sim GM\right)}{P\left(\sim LC\right)}=\frac{0.4\times 0.2}{0.1}=0.08,$$

$$P\left(\sim DS,GM|\sim LC\right)=\frac{P\left(\sim LC|\sim DS,\;GM\right)P\left(\sim DS,GM\right)}{P\left(\sim LC\right)}=\frac{0.5\times 0.1}{0.1}=0.05,$$

$$P\left(\sim DS,\sim GM|\sim LC\right)=\frac{P\left(\sim LC|\sim DS,\sim GM\right)P\left(\sim DS,\sim GM\right)}{P\left(\sim LC\right)}=\frac{0.2\times 0.1}{0.92}=0.02$$
